# Artificial light and neurodegeneration: does light pollution impact the development of Alzheimer’s disease?

**DOI:** 10.1007/s11357-023-00932-0

**Published:** 2023-09-21

**Authors:** Julia Karska, Szymon Kowalski, Anna Gładka, Anna Brzecka, Marta Sochocka, Donata Kurpas, Jan Aleksander Beszłej, Jerzy Leszek

**Affiliations:** 1 Department of Psychiatry, Wrocław Medical University, Pasteura 10, 50-367 Wrocław, Poland; 2 Faculty of Medicine, Wrocław Medical University, Pasteura 1, 50-367 Wrocław, Poland; 3 Department of Pulmonology and Lung Oncology, Wrocław Medical University, Grabiszyńska 105, 53-439 Wrocław, Poland; 4 Hirszfeld Institute of Immunology and Experimental Therapy, Polish Academy of Sciences, Rudolfa Weigla 12, 53-114 Wroclaw, Poland; 5 Health Sciences Faculty, Wroclaw Medical University, Bartla 5, 50-996 Wrocław, Poland

**Keywords:** Light pollution, Neurodegeneration, Alzheimer’s disease, Dementia, Circadian rhythm

## Abstract

Two multidimensional problems of recent times — Alzheimer’s disease and light pollution — seem to be more interrelated than previously expected. A series of studies in years explore the pathogenesis and the course of Alzheimer’s disease, yet the mechanisms underlying this pathology remain not fully discovered and understood. Artificial lights which accompany civilization on a daily basis appear to have more detrimental effects on both environment and human health than previously anticipated. Circadian rhythm is affected by inappropriate lighting conditions in particular. The consequences are dysregulation of the sleep-wake cycle, gene expression, neuronal restructuring, brain’s electricity, blood flow, metabolites’ turnover, and gut microbiota as well. All these phenomena may contribute to neurodegeneration and consequently Alzheimer’s disease. There is an increasing number of research underlining the complexity of the correlation between light pollution and Alzheimer’s disease; however, additional studies to enhance the key tenets are required for a better understanding of this relationship.

## Introduction

The development of industry results in many environmental changes that can have a potentially negative impact on health. Light pollution is one of such industrialization’s side effects. About 80% of the population worldwide is influenced by excessive amounts of unnatural light [1]. Following the International Dark Sky Association, light pollution is the inappropriate or excessive use of artificial light that may cause serious environmental consequences for humans, wildlife, and climate. Most studies explore the influence of light at night and blue light on human health [2–5]. It is investigated that light pollution could affect many systems of the human body, and data indicate its indirect positive association with retina pathologies, cardiovascular damage, depression, cancer, and sleep disturbances [3, 6]. The latter shows the possible association with triggering neurodegeneration since sleep is essential for neurons to regenerate their plasticity and remove toxic compounds. In light pollution and its impact on neurodegeneration, the role of outdoor and indoor dim artificial light at night (dLAN) is noted [7, 8]. The dLAN strength is usually 5–10 lux which is also said to be the minimum quantity of light pollution in many countries [9]. It is discussed whether Alzheimer’s disease (AD) as a neurodegenerative disease may be induced by inappropriate artificial light [7, 8]. AD is one of the most common types of dementia and neurodegenerative disorders. Around the world, 55 million people have dementia, which will increase by 78 million in 2030 and 139 million in 2050 according to WHO [10]. Considering the significance of light pollution and AD, this review highlights the potential and multifaceted link between these two problems.

## Light pollution’s influence on molecular mechanisms of neurodegeneration

An increasing number of studies emphasize the essential role of light in the course of neurodegenerative diseases (Table 1). The deregulation of circadian rhythms’ mechanisms may be responsible for this influence.

**Table 1:** Research concerning the relationship between molecular aspects of circadian rhythm disruption and neurodegeneration

Author, year, citation	Type of study	Group	Main findings
Namgyal D. et al. 2020 [9]	Experimental study	Swiss Albino mice exposed to dim light at night (dLAN) for 3 weeks	Modulation of hippocampal protein expression coded by genes Brain-Derived Neurotrophic Factor (BDNF), cAMP Response Element-Binding Protein (CREB), Doublecortin (DCX), Synapsin (SYN), and Sirtuin 1 (SIRT1) causes downregulation of CREB and SIRT1 mRNAs and neurodegeneration-associated miRNA21a-5p and miRNA34a-5p
Shi L. et al. 2018 [10]	Meta-analysis	-	Individuals with sleep disorders have a 1.49-fold increased risk of AD as compared to subjects with no sleep disruptions
Musiek E. S. et al. 2013 [11]	Experimental study	Mice	Reduction of expression of BMAL1 (Basic Helix-Loop-Helix ARNT Like 1) gene in the cortex and hippocampus induced severe reactive astrocytosis, neuronal oxidative damages, and the degeneration of synaptic terminals
Nash TR et al. 2019 [12]	Experimental study	Flies maintained in daily cycles of 12-h blue LED and 12-h darkness or in constant darkness or in white light with blocked blue wave exposure	Group maintained in daily cycles of 12-h blue LED and 12-h darkness had significantly reduced length of life compared with other groups. The blue-light exposure accelerated aging phenotypes, damage retinal cells, cause brain neurodegeneration, and impair locomotion
Kang J. E. et al 2009 [13]	Experimental study	In vivo microdialysis in mice	Amyloid β (Aβ) levels in cerebrospinal fluid (CSF) have a diurnal pattern and are correlated with wakefulness
Tarasoff-Conway J. M. et al. 2015 [14]	Review	-	Impairment of the clearance of Aβ and tau out of the brain parenchyma under sleep deprivation
Li Y. et al. 2020 [15]	Review	-	Melatonin decreases Aβ-induced neurotoxicity and probably improves Aβ clearance
Kim M. et al. 2018 [16]	Experimental study	Tauopathy/AD flies exposed to dLAN for 3 days	Increased number of pTau proteins and neurodegeneration level was related partially to the altered circadian rhythm
Hui CK et al. 2023 [17]	Experimental study	Zebra finches exposed to dim artificial light at night (ALAN) of 1.5 lux	Immediate early gene expression as a proxy of brain activity related to the response to dim ALAN expression in birds exposed to ALAN was significantly different from birds inactive at night. The changes in expression concerned several brain included areas associated with memory

### The physiological function and disruption of the circadian rhythm

Complex circadian timing systems are essential in most physiological processes in mammals, including sleep [8, 18]. The crucial elements of the molecular clock are remarkably evolutionary conserved, which might be a cause of their fundamental significance for life [8, 18]. The master pacemaker, located in the ventral hypothalamus’s suprachiasmatic nucleus (SCN), can synchronize complementary data from peripheral cells. The phase of the master clock must be periodically readjusted by light signals to keep it aligned with real geophysical time [8, 18]. In both SCN neurons and peripheral cells, the circadian clockwork is constructed from an interactive network of transcriptional and translational loops. The metabolism of neurotransmitters which take part in the mammalian circadian oscillator is regulated by the transcription factors such as albumin D-site-binding protein (DPB), hepatic leukemia factor (HLF), and thyrotroph embryonic factor (TEF). The system of circadian loops has yet to be fully discovered [19].

The micro and macro worlds follow the circadian rhythm of light and darkness. The development of artificial light sources disrupted the functioning of the inner clock. As a result, one of the most severe environmental threats may be light pollution, which could lead to chronic circadian desynchrony [8, 20].

Lucassen et al. investigated the health impact of continuous exposure to light. Mice were exposed to continuous light for 24 weeks, then major health parameters were measured. It has been shown that rhythmicity in the central circadian pacemaker of SCN was significantly reduced. Furthermore, reduction of skeletal muscle function, trabecular bone deterioration, and induction of a transient pro-inflammatory state have been observed. Research finally showed that after the mice were returned to a light-dark cycle, the SCN neurons recovered their normal high-amplitude rhythm, and the measured health parameters returned to normal [21]. The other study has determined that flies (*drosophila melanogaster*) maintained in daily cycles of 12-h blue LED and 12-h darkness had significantly reduced length of life compared with flies maintained in constant darkness or white light with blocked blue wave exposure. Moreover, it has been shown that blue-light exposure can accelerate aging phenotypes, damage retinal cells, cause brain neurodegeneration, and impair locomotion [12].

Evidence suggests that circadian rhythm disruption and connected sleep deprivation could be crucial risk factors for the development of AD [22–24]. Such individuals have a 1.49-fold higher risk of AD than subjects with no sleep and circadian disruptions [10]. It is suggested that the problem of light pollution may contribute to the relationship between artificial light and the development of neurodegenerative diseases.

### Melatonin

A disrupted melatonin-releasing pattern could also play an important role in AD pathogenesis. This pineal gland’s hormone modulates the regulatory network of secretase expression and function [15, 25]. That may inhibit the amyloid precursor protein (APP) processing and Aβ production. Melatonin decreases Aβ-induced neurotoxicity and probably improves Aβ clearance via glymphatic-lymphatic and degradation pathways [15, 25]. It is hypothesized that melatonin restores cholinergic neurotransmission typically disrupted in AD [26]. The suggested mechanism inhibits the calcium-induced acetylcholinesterase (AChE) release, which may enhance acetylcholine acting [27]. Furthermore, melatonin is also recommended to alleviate the altered glutamatergic system in AD via inhibition of the N-methyl-D-aspartate receptor (NMDA) receptors [28]. Hypothetically, melatonin or potential melatonin receptor agonists could be a promising tool to prevent the accumulation of pathological proteins in AD [15, 25]. It is indicated in a recent meta-analysis that AD patients receiving >12 weeks of melatonin treatment improve their Mini Mental Scale Examination score [29].

### Mechanisms of the influence of artificial light on the human organism

Two basic mechanisms are involved in adaptation to artificial light: image forming (IF) for vision and non-image forming (NIF) adaptation of physiology and behavior to light. NIF photoresponses are very varied and associated with the adjustment of pupil diameter, but also with slower responses, such as adaptation of the circadian clock to the daily day-night cycle. The cells responsible for NIF responses produce photopigment melanopsin and they are known as intrinsically photosensitive retinal ganglion cells (ipRGCs) [30, 31]. The role of ipRGCs is the integration of the pathways of phototransduction derived from the rod/cone and melanopsin actions. ipRGCs affect the SCN and other regions, including the intergeniculate leaflet of the thalamus, the olivary pretectal nucleus controlling pupil constriction, and structures engaged in emotions, cognition, or memory [32]. Important to highlight is that NIF responses have been found almost intact in experimental animals and blind human patients with complete degeneration of rod/cone photoreceptors [33, 34].

The invention of efficient and economically practical blue light-emitting diode (LED) changed daily lighting worldwide. The majority of the LED spectrum is blue light with 460 nm and this fraction is higher than in any other incandescent light [35]. The spectrum of ipRGCs and melanopsin action is the highest at 480 nm — the blue portion of light. Therefore, it is essential for NIF functions throughout the day and circadian synchronization. Inappropriate timing, intensity, or duration of blue light may yet result in circadian phase delay, melatonin suppression, sleep quality alteration, or reduction of cognitive performance [36]. It has been reported that the blue component of light could have a phototoxic effect on the retina since blue light presents the highest photon energy of the visible spectrum [37].

### Circadian rhythm and CSF clearance from AD’s pathological proteins

**Fig. 1:**
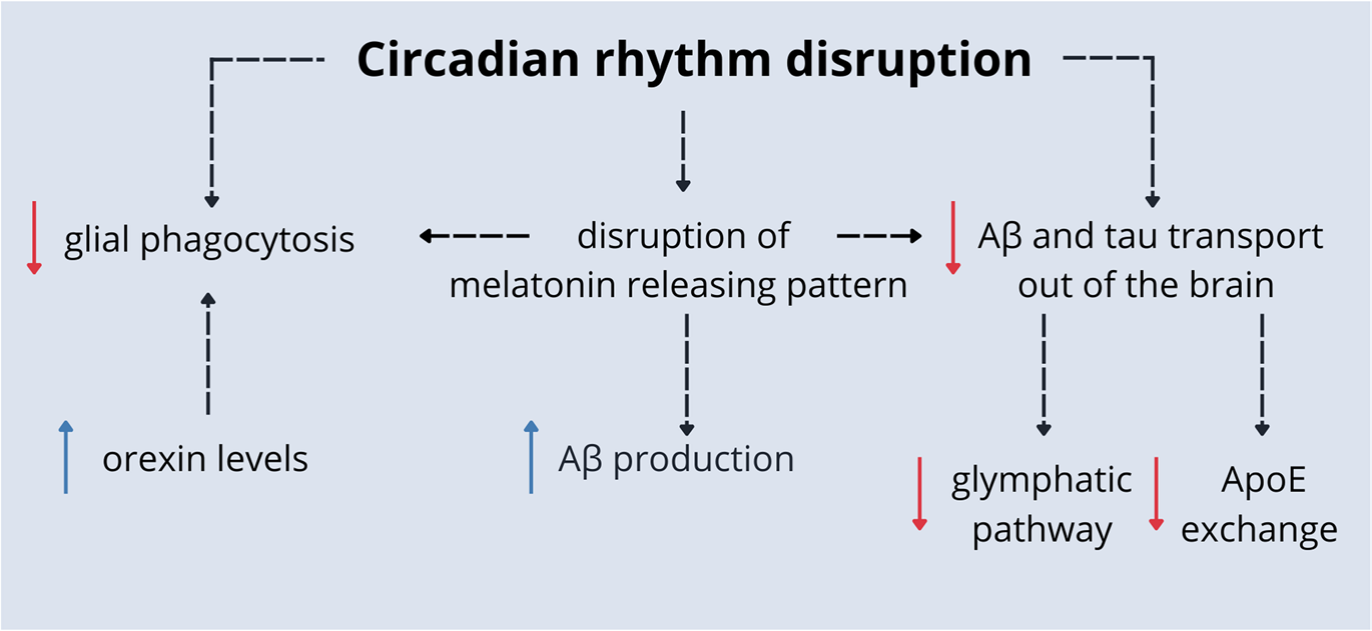
The potential mechanisms of AD development. Under conditions of circadian rhythm disruption, there is an accumulation of neurotoxic proteins that are not removed from brain tissue via glial phagocytosis or active transport. These processes are intensified by high orexin levels and disrupted melatonin releasing patterns. Moreover, impairment of melatonin release leads to more excellent production of Aβ [13, 15, 24, 25, 38]

Both sleep duration and quality are associated with Aβ cerebrospinal fluid (CSF) or interstitial fluid (ISF) concentration. Aβ levels in CSF show a diurnal pattern, increasing throughout the day, peaking at night, and decreasing overnight. It is suggested that the duration of wakefulness, instead of the circadian time point, is the factor affecting ISF-Aβ concentration [13]. The knowledge about the role of sleep and circadian rhythms in the brain protein clearance system is constantly increasing. In physiological conditions, Aβ and tau are either degraded by proteases or glial phagocytosis, or they may be transported out of the brain via ISF-CSF exchange by the glymphatic pathway or ISF-blood exchange by apolipoprotein E (ApoE) [14]. Under sleep deprivation, increased noradrenaline stimulation leads to swelling of the neurons. As a result of increased resistance, the CSF-ISF bulk flow is disrupted, inhibiting the delivery of ApoE. There is also a disturbance of the glymphatic pathway. These processes lead to impairment of the clearance of Aβ and tau out of the brain parenchyma. Furthermore, in prolonged waking and circadian rhythm disruption, orexin level keeps elevated and impairs phagocytic clearance of Aβ and tau [13, 24, 38] (Fig. 1).

### Genetic aspects

Circadian rhythm disturbances connected with artificial light exposure may lead to neurodegenerative processes on the genetic level [9].

It was explored in a study performed on mice exposed to dLAN for 3 weeks. It led to the modulation of hippocampal protein expression coded by genes known to be associated with neurodegeneration — *BDNF*, *CREB*, *DCX*, *SYN*, and *SIRT1.* Disturbances in cognitive behavior occurred as a consequence of the exposure [9]. The additional downregulation of neurodegeneration-associated miRNA21a-5p and miRNA34a-5p could contribute to the abnormal behavior presented by mice [9].

Hui et al. exposed zebra finches to dim artificial light at night (ALAN) of 1.5 lux and analyzed 24 regions of the brain. In this study, the overall expression of two different IEGs (immediate early genes as a proxy of brain activity related to the response to dim ALAN) cFos and ZENK was used. IEG expression in birds exposed to ALAN was significantly different from birds inactive at night. The changes in expression concerned several brain areas associated with vision, movement, learning and memory, pain processing, and hormone regulation [17].

**Fig. 2:**
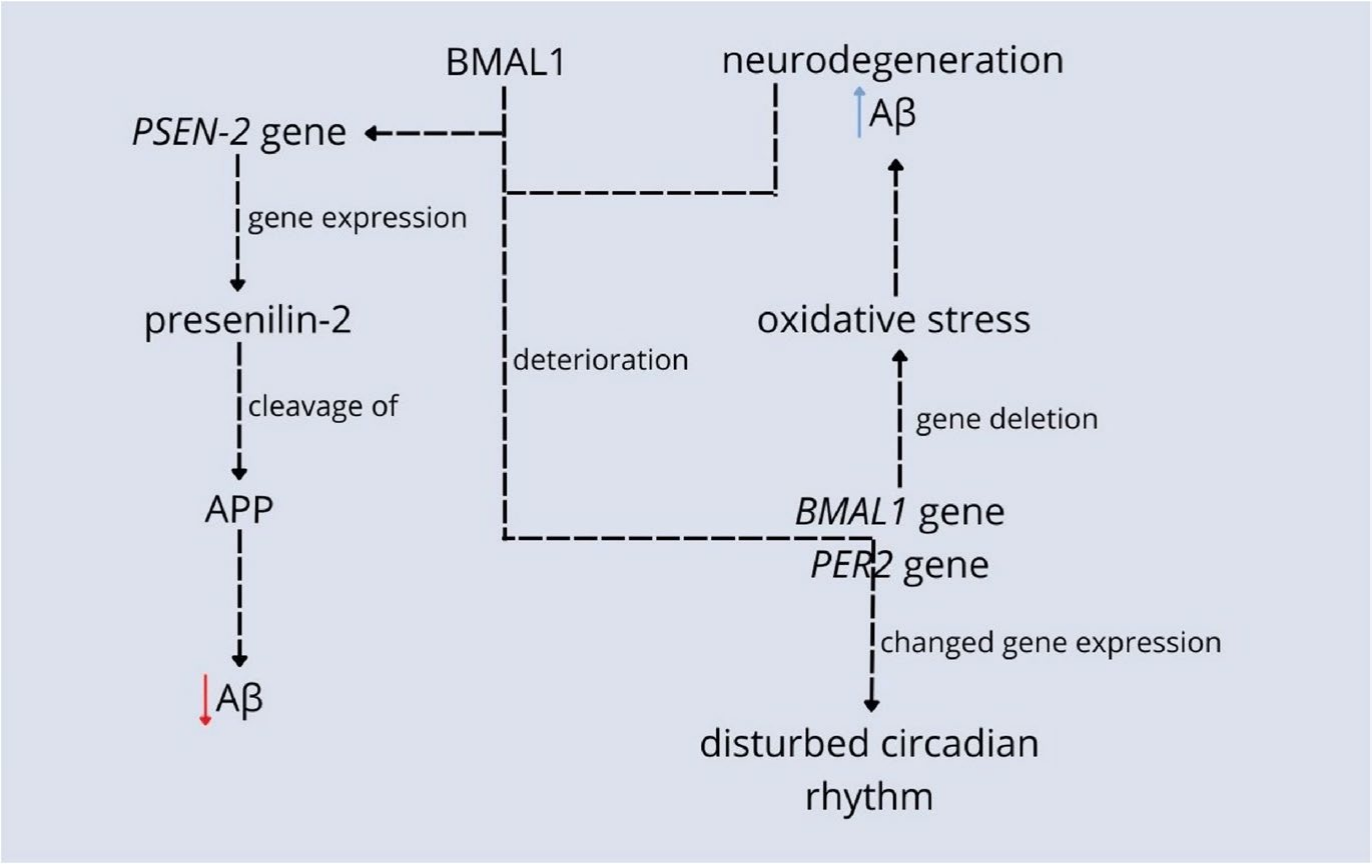
Relations between genes responsible for circadian rhythm and neurodegeneration are numerous. Deleting the *BMAL1* center clock’s gene in the cortex and hippocampus leads to oxidative stress including severe reactive astrocytosis, neuronal oxidative damage, the degeneration of synaptic terminals, and neurodegeneration. In turn, neurodegenerative protein Aβ triggers deterioration of BMAL1 and CBP center clock proteins which then change the expression of *BMAL1* and *PER2* center clock’s genes resulting in a disturbed circadian rhythm. BMAL1 and CLOCK proteins also activate *PSEN-2* gene expression which codes presenilin-2 protein. This protein cleaves APP and, as a result, diminishes the amount of Aβ [11, 22, 39, 40]

The circadian rhythm disruption may be caused by the deposition of pathological proteins in the brain [39]. The possible explanation of this relation is that Aβ triggers the deterioration of circadian clock proteins BMAL1 and CREB-binding Protein (CBP), causing changes in the expression of circadian clock genes *BMAL1* and *Period Circadian Regulator 2 (PER2)* with consequences of a disturbed sleep-wake cycle [39]. Recent studies also suggest that the accumulation of tau aggregates is related to decreased nonrapid eye movement (NREM) sleep slow wave activity [40]. In the presence of tau, cyclic expression of the circadian clock proteins PER2 and BMALI1 is interrupted in both the hippocampus and the hypothalamus, confirming tau’s detrimental effect on the circadian rhythm [22]. Experimental deletion and reduction of expression of the *BMAL1* gene in the cortex and hippocampus induced severe reactive astrocytosis, neuronal oxidative damages, and the degeneration of synaptic terminals. It suggests that changed circadian functions could be responsible for developing neurodegeneration through lowered BMAL1 [11] (Fig. 2).

Defensive mechanisms of Aβ clearance in CSF are also related to the circadian clock’s genes. The presenile-2 protein (PSEN-2) is expressed in the presence of clock genes *Clock Circadian Regulator (CLOCK)* and *BMAL1*. PSEN-2 is responsible for the cleavage of, e.g., Aβ and thus diminishes its level in the brain. In the hereditary type of AD, the *PSEN-2* gene is muted, resulting in a reverse effect than in the physiological state, accumulation of Aβ, and neurodegeneration [41, 42] (Fig. 2).

One in 3 days of exposure to dLAN may promote neurodegeneration in AD models. According to this study, the tauopathy/AD flies after dLAN exposure presented an altered circadian rhythm, which led to an increased number of pTau proteins and neurodegeneration levels [16]. It was then suggested that dLAN could exacerbate neurodegenerative disease, including AD, among AD model, flies [16].

## The indirect influence of light pollution on AD and other neurodegenerative diseases

Light pollution may affect neurodegeneration through changes in levels of lipids in the blood, vessel structure, or gut microbiota among others. It is suggested that artificial light also impacts neurodegenerative diseases other than AD (Table 2).

**Table 2:** Studies concerning the indirect relationship between light pollution and neurodegeneration

Author, year, citation	Type of study	Group	Main findings
Chen Y. et al. 2022 [7]	Observational study	Chinese veterans exposed to long-term outdoor dLAN	Females and those with lower educational or social activity level have higher risk of developing mild cognitive impairment (MCI)
Habert M. O. et al. 2011 [43]	Prospective, longitudinal, multi-centric study	Patients with AD/MCI	Hippocampal hypoperfusion in patients with MCI/AD, possibly related to circadian changes in locomotor activity or blood pressure
Liu P. et al. 2019 [44]	Cohort study	Patients with AD	The composition of gut microbiota in AD patients seems to resemble the microbiome after changes in circadian rhythm
Hamilton R. L. 2000 [45]	Cohort study	145 sporadic AD cases	Lewy bodies may exist in brains of 60% of AD sporadic individuals, potential role of circadian disfunction
Wright Willis A. et al. 2010 [46]	Observational study	Prevalence of Parkinson’s disease (PD) according to satellite-observed sky light pollution	Prevalence of PD when age- and race-adjusted is significantly correlated with average satellite-observed sky light pollution
Romeo S. et al. 2013 [47]	Experimental study	Rats exposed to the bright light (3000 lux)	Higher level of euromelatonin neurons and lower level of tyrosine hydroxylase (TH)-positive neurons in the substantia nigra
Wang H. 2015 [48]	Experimental study	Huntington’s disease (HD) mice models exposed to dLAN (20 lux)	Worsening of HD symptoms due to dysregulated sleep-wake cycle
Xiang Xu Y. et al. 2022 [49]	Observational study	Chinese teenagers exposed to post-bedtime and pre-awake dLAN	Light after bedtime was related to elevated fasting insulin and HOMA-IR while pre-awake light to elevated total cholesterol, triglyceride, and low-density lipoprotein cholesterol

### Obesity and cardiovascular system

Indirect influences of light could explain the relation between light pollution and neurodegeneration. Research suggests that light pollution may contribute to obesity and overall higher cardiometabolic risk. Geographically and historically, there is a coincidence between the appearance of obesity and the availability of artificial light. Experimental studies on both animals and humans underline the role of disturbances in the circadian rhythm as a convergence between light pollution and obesity [8]. The association between light, circadian rhythm, and cardiometabolic risk in turn was observed in the study on the effects of post-bedtime and pre-awake dLAN in the bedrooms of Chinese teenagers. It presented that exposure to light after bedtime might be related to elevated fasting insulin and HOMA-IR (Homeostatic Model Assessment for Insulin Resistance) while pre-awake light to elevated total cholesterol, triglyceride, and low-density lipoprotein cholesterol (LDL) [49]. It is well-known and confirmed in multiple studies that cardiometabolic risk factors, including obesity, support neurodegeneration [50, 51]. It could be then suggested that light pollution through interruption of the sleep-wake cycle and metabolic changes consequently may contribute to the development of neurodegenerative disorders, including AD.

Vascular dysfunction is another aspect that connects circadian rhythm and AD. It occurs that human cerebral blood flow velocity is under the control of circadian rhythm, but it is independent of circadian changes in locomotor activity or blood pressure [52]. The risk of AD is characterized by hypometabolism and cerebral hypoperfusion as well [43]. Hypothetically, alterations in circadian rhythm may cause vascular and blood flow changes that contribute to AD development, yet it must be fully confirmed.

### Microbiota

There is also growing evidence of the role of gut microbiota in the occurrence of AD. The circadian rhythm seems to regulate the gut microbiota. Circadian functions when disturbed could induce changes in microbiota structure. This bacterial alteration, especially the loss of intestinal microbiota, may lead to the greater permeability of the gut barrier and consequently systemic inflammation with impairment in the blood-brain barrier and neuroinflammation as well [53–55]. Furthermore, the composition of gut microbiota in AD patients resembles the microbiome after changes in circadian rhythm [44].

### Other types of dementia

MCI as a state between normal cognitive aging and early dementia as well as a known risk factor for AD seems to also be triggered by dLAN. It has been discovered that Chinese veterans, exposed for a long term to outdoor dLAN, presented a higher risk of developing MCI. This might suggest that light pollution in some cases plays a role in developing AD from the earliest stages [7].

Studies indicate that the development of AD could be related to dementia with Lewy bodies (DLB) pathogenesis with the role of a disrupted sleep-wake cycle. Since Lewy bodies may exist in the brains of 60% of AD sporadic individuals, it is possible that also through these neurodegenerative structures, circadian dysfunction contributes to the course of AD [45, 56, 57].

Studies also assessed the influence of light pollution on neurodegenerative diseases other than AD. Such a relation is determined for both Parkinson’s (PD) and Huntington’s disease (HD) so far. The prevalence of PD when age- and race-adjusted is significantly correlated with average satellite-observed skylight pollution [46]. Another study might explain this correlation in the molecular aspects. Rats exposed to bright light (3000 lux) presented higher levels of neuromelatonin neurons and lower levels of tyrosine hydroxylase (TH)-positive neurons in the *substantia nigra* which suggested oxidative stress in this region caused by light [47]. In HD, the detrimental effect of the light may be associated with disrupting the circadian rhythm. HD mice models exposed to dLAN (20 lux) show worsening HD symptoms due to dysregulated sleep-wake cycle [48].

## Potential problem-solving strategies

Efforts are underway to design our living areas with lighting that is more compatible with our biology. This is beneficial from the socio-economic, ecological, and health perspectives [58]. However, until some of these practices become widespread, individuals are still exposed to environmental light pollution. Nevertheless, daily routine changes and indoor light exposure are significant. It was shown that the bright light presence in the morning and reduced light exposure during the evenings could increase sleep quality and reduce agitation in patients with dementia [59–61].

Another aspect of this topic is that in many electrical light bulbs used today and considered “environmentally friendly,” such as light-emitting diodes (LEDs) devices, electrical energy is converted into short-wavelength illumination. LEDs illuminate industrial environments in TVs, computers, smartphones, and tablets. Although the light emitted by most LEDs appears white, LEDs have peak emission in the blue light range (400–490 nm), which could be one of the leading causes of disruption of circadian rhythms [62]. Moreover, studies show that blue light may decrease human melatonin levels and cause response delays and depressive-like emotions in mice [63, 64]. In this case, a healthier alternative may be a blue-free WLED (white light-emitting diode) that can avoid chronodisruption [20].

Environmental and technological light pollution leads to disturbances in the functioning of the biological clock and its subsequent effects. Therefore, it is possible that the circadian rhythm’s normalizing, especially rest-activity patterns, will benefit people with AD. In that case, bright light therapy seems an exciting tool, often administered to treat circadian rhythm disturbances [23, 65, 66]. It is shown that appropriate protocols of bright light therapy can positively affect cognitive functions, length, and quality of sleep, reduce symptoms of depression, and improve appetite in patients with dementia [67, 68]. These results pave the way for further research and the creation of new optimal therapeutic protocols. Moreover, the positive effects of using the optimal lighting pattern could be used in facilities that care for people with AD [65].

## Final remarks and conclusions

dLAN seems to be the most important type of light pollution that may contribute to neurodegeneration. Exposure to dLAN could lead to the modulation of hippocampal protein expression and the intensification of oxidative stress, sleep disturbances, and dysregulation of the biological clock [9, 16, 69–71]. Under circadian rhythm disruption, there is a more significant accumulation of neurotoxic proteins such as tau or Aβ. These proteins are not adequately removed from the brain via glial phagocytosis or active transport [13, 24, 25, 38, 42]. The impact of circadian rhythm disruption on BMI, vascular disorders, gut microbiota dysfunction, and neuroinflammation could take part in the pathogenesis of the AD [43, 44, 52, 54, 55]. Therefore, it seems possible that normalizing the rest-activity pattern, through the reduction of light pollution, among others, would be one of the protective factors in the development of neurodegeneration. Examples of such actions include using appropriate artificial lighting patterns or bright light therapy [20, 66].

Additional studies to understand more completely the key tenets of light pollution on human health are required. Especially the long-term effects of extensive blue light in LEDs on the human body remain to be analyzed. It needs be noted that research should contain not only light intensity but also its wavelength as well as temporal and spatial resolution in light measurements. The mechanistic aspects of light pollution’s influence on the body’s functioning should also be explored in detail in future studies. Possibly more research on humans instead of animal models would present more relevant results in the context of medicine, so far deficiency in the scientific literature.

The extent and intensity of artificial night lighting increase so much that it has substantial effects on the biology of individuals, leading to the development of various pathological conditions. A lot of indirect evidence indicates that light pollution can affect the development of many neurodegenerative diseases, including AD. More extensive broad-spectrum studies are needed for a better understanding of the multifaceted connection between AD and light pollution to create a balanced model of everyday artificial lightning.
